# Mobile opportunity against stress: Open study protocol on the effectiveness of a mobile platform for stress self-management in the post-pandemic era

**DOI:** 10.3389/fpsyg.2022.917574

**Published:** 2023-01-23

**Authors:** Andrea Pozza, Barbara Giangrasso, David Baldo, Ada Fort, Giovanna Millozzi, Francesco Iocca, Nicole Loren Angelo, Daniele Pugi, Giacomo Gualtieri, Lore Lorenzi, Francesca Gioia, Sara Bocci Benucci, Giulia Fioravanti, Anna Coluccia, Fabio Ferretti, Silvia Casale

**Affiliations:** ^1^Department of Medical Sciences, Surgery and Neurosciences, University of Siena, Siena, Italy; ^2^Department of Health Sciences, Psychology Unit, University of Florence, Florence, Italy; ^3^Department of Information Engineering and Mathematics, University of Siena, Siena, Italy; ^4^Santa Maria Alle Scotte University Hospital of Siena, Siena, Italy

**Keywords:** digital health, mental health, stress, smartphone apps, pandemic (COVID-19)

## Abstract

Mobile health platforms have shown promise in the management of various mental health conditions (including stress, anxiety, and depression) and cognitive behavioral strategies emerged as a popular and effective option offered by the platforms. This paper presents the protocol of a study aimed to test the effectiveness of a mobile platform that uses cognitive-behavioral strategies for stress self-management in the Tuscany region (Italy). The mobile app is adapted to the specific needs of each vulnerable population for which it is designed: young and older people, healthcare professionals, entrepreneurs. The app will be evaluated on the following outcomes: (i) perceived susceptibility and severity of the pandemic situation, perceived benefits, and costs of preventive health behaviors, (ii) knowledge about Covid-19 preventive behaviors and negative consequences of social distancing, (iii) stress and psychopathological symptoms (i.e., anxiety, depression, and post-traumatic stress symptoms) and cognitive distortions. If successful, we expect that the platform could give various groups clinical benefits by providing symptom self-monitoring and early intervention, consolidating the number of mental health programs available, and decreasing barriers to treatment-seeking. This population-level approach has the potential to improve mental health outcomes in pandemic periods for many people.

## Introduction

### Background

The Covid-19 pandemic has caused a worldwide health crisis. Italy was the first western country to be affected by Covid-19 and, since the first confirmed case on February 20, 2020, this disease dramatically impacted the Italian society. Indeed, in March 2020, the Italian government established extraordinary preventive measures such as quarantine, lockdown, and physical distancing (Italian Ministry of Health, 2020). Gradually, thanks to the vaccination campaign, pandemic-induced stress regarding risk of infection, inadequate access to protective devices, and prolonged social isolation mitigated and the spread of the infection decreased (Davico et al., 2021; Prati and Mancini, 2021; Medda et al., 2022). The Covid-19 pandemic has had a significant impact on people’s everyday life and negative implications for mental health in the general population. In particular, low mood, irritability, anger, insomnia, emotional exhaustion, depression, anxiety, and stress have been variably but largely reported during the outbreak of Covid-19 all over the world (i.e., Brooks et al., 2020; Mazza et al., 2020; Odriozola-González et al., 2020; Salari et al., 2020; Pan et al., 2021; Wang et al., 2021; Medda et al., 2022). Locations hit hardest by the pandemic in 2020 had the greatest increases in prevalence of depressive and anxiety disorders (COVID-19 Mental Disorders Collaborators, 2021). Despite a wide and varying prevalence of mental health problems and psychosocial consequences across countries and regions, as Nochaiwong et al. (2021) highlighted, the global prevalence of depression was 28.0%, anxiety was 26.9%, and the rate of stress was 36.5%. The pandemic accounted for a global increase of 27.6 and 25.6% of cases of major depressive disorder and anxiety disorders, respectively. Daily Covid-19 infection rates and reductions in human mobility were associated with increased prevalence of major depressive disorder and anxiety disorders (COVID-19 Mental Disorders Collaborators, 2021). Levels of anxious-depressive symptoms were lowest when covid-19-related restrictions were lifted and highest when restrictions were in place (Moulin et al., 2023). Consequently, implementing empirically supported strategies to assist in managing stress and minimizing concomitant mental health problems has become a priority.

## Vulnerable/at risk populations for COVID-19 related psychological distress

Certain risk factors for the development of Covid-19-related psychological distress and other psychological symptoms have been found (for a review see Brooks et al., 2020). It has been shown that economic (i.e., unemployment, loss of job) and social (i.e., social distance, mandatory use of the masks, online learning) factors are strongly associated with Covid-19-related distress among young people, despite their relatively low risk of virus-related health complications (Jung et al., 2020; McGinty et al., 2020; Pierce et al., 2020; Roma et al., 2020; Shanahan et al., 2020; Rossell et al., 2021; Varma et al., 2021). Evidence also suggests that older adults – particularly those with previous comorbid health conditions or cognitive impairment – might be at risk of psychological symptoms due to prolonged isolation and anxiety about infection risk (Chong et al., 2020; Forlenza and Stella, 2020; Girdhar et al., 2020; Peyman and Olyani, 2020; Roma et al., 2020). Moreover, they are expected to adhere to preventive measures and restrictions for extended periods, to reduce the risk of contracting the infection. Healthcare workers also deserve special attention as a potential vulnerable population (Naushad et al., 2019; Brooks et al., 2020; Mahmud et al., 2021) as during the Covid-19 pandemic they have had to cope with an increasing workload, fear of infection, contagion and transmission to their families, frustration, highly challenging decisions, guilt and bereavement, intrusive thoughts, nightmares, physical exhaustion, and inadequate personal equipment (Elbay et al., 2020; Holmes et al., 2020; Krystal, 2020; Lasalvia et al., 2021). These experiences have impacted their mental health leading to symptoms of depression, anxiety, and distress (Kisely et al., 2020; Pappa et al., 2020; De Kock et al., 2021; Mahmud et al., 2021). In fact, when assessed with psychiatric diagnostic interviews during the pandemic, health care workers reported a prevalence of 14.3% for Generalized Anxiety Disorder, 13.7% for Depression, and 7.9% for Post-Traumatic Stress Disorder (PTSD). These prevalence estimates were lower when compared to screening instruments (Scott et al., 2023). Although the occurrence of mental health symptoms remained equally high between the first (March until June 2020) and the second surge (October 2020 to June 2021), Intensive Care Unit (ICU) nurses were more likely to experience work-related fatigue (Heesakkers et al., 2023).

Finally, the economic impact has been likewise devastating, forcing entire professional sectors (such as tourism, hotels, sports, entertainment, restaurant) to stop working. In this regard, entrepreneurs might represent another underestimated vulnerable population (Patel and Rietveld, 2020; Weems et al., 2020; Grandi et al., 2022). Therefore, the emotional responses triggered by the pandemic and their management appear substantial among vulnerable groups, such as young adults, older people, frontline and non-frontline healthcare workers, and entrepreneurs (Ho et al., 2020; Qiu et al., 2020; Trumello et al., 2020; Lasalvia et al., 2021; Wang et al., 2021).

## Theory-driven/evidence-based interventions for reducing psychological distress during COVID-19 pandemic

During the Covid-19 pandemic, the Health Beliefs Model (HBM; Becker and Maiman, 1975) has been found to be effective in promoting preventive health behaviors (Chen et al., 2019; Mukhtar, 2020; Tong et al., 2020; Carico Jr. et al., 2021; Jose et al., 2021; Wong et al., 2021) including hand washing, face mask wearing, maintaining social distance (Li et al., 2021), and receiving vaccination (Hossain et al., 2021). The HBM consists of six main aspects: (i) perceived susceptibility, (ii) perceived severity of getting an infection and perceived likelihood of disease transmission, (iii) perceived benefits of taking action to reduce the risk or seriousness of impacts, (iv) perceived barriers to taking action such as tangible and psychological costs of the advised action, (v) cues to actions or strategies to enhance one’s responsiveness, and (vi) individual, demographic, and psychosocial characteristics, which can affect perceptions of health-related behaviors (Janz and Becker, 1984; Kim et al., 2012; Chen et al., 2019; Jose et al., 2021). Overall, HMB-based interventions mitigate behaviors influenced by perceived health threats which provoke anxiety and fear, reinforcing individuals’ perceived benefits and self-efficacy (Janz and Becker, 1984; Kim et al., 2012).

Among the evidence-based strategies, Cognitive Behavioral Therapy (CBT) techniques for reducing Covid-19 related psychological distress have shown promising results (Aminoff et al., 2021; Sharrock et al., 2021; Song et al., 2021). Within the CBT framework, unhelpful ways of thinking and cognitive distortions are both risk and maintaining factors for psychological and psychiatric symptoms (Beck, 1963, 1979; Taylor, 2019). More specifically, people normally experience events according to their personal core beliefs which in turn lead to automatic thoughts, and typical emotional and behavioral responses. In case of negative core beliefs and subsequent negative automatic thoughts, any sort of event might result in negative emotions and maladaptive behaviors, maintaining poor mental states (Burns, 1980; Mathews et al., 1997; Rnic et al., 2016). Common cognitive distortions include *catastrophizing, overgeneralization*, and *minimizing the positive* (Burns, 1980).

## Use of mobile technology for health interventions

Mobile technology represents an available and attractive channel for health interventions (Donker et al., 2013; Beiwinkel et al., 2017). Digital health technologies have been utilized to manage numerous psychological symptoms such as depression, anxiety, and stress, major mood disorders, substance use, eating disorders, self-harm, and suicidal ideation (Witt et al., 2017; Hwang and Jo, 2019; Storm et al., 2021; Torous et al., 2021; Amanvermez et al., 2022); nonetheless, therapist supported/guided or blended interventions appeared more effective than unguided approaches (Harrer et al., 2018; Linardon et al., 2019; Taylor et al., 2021).

**Figure 1:**
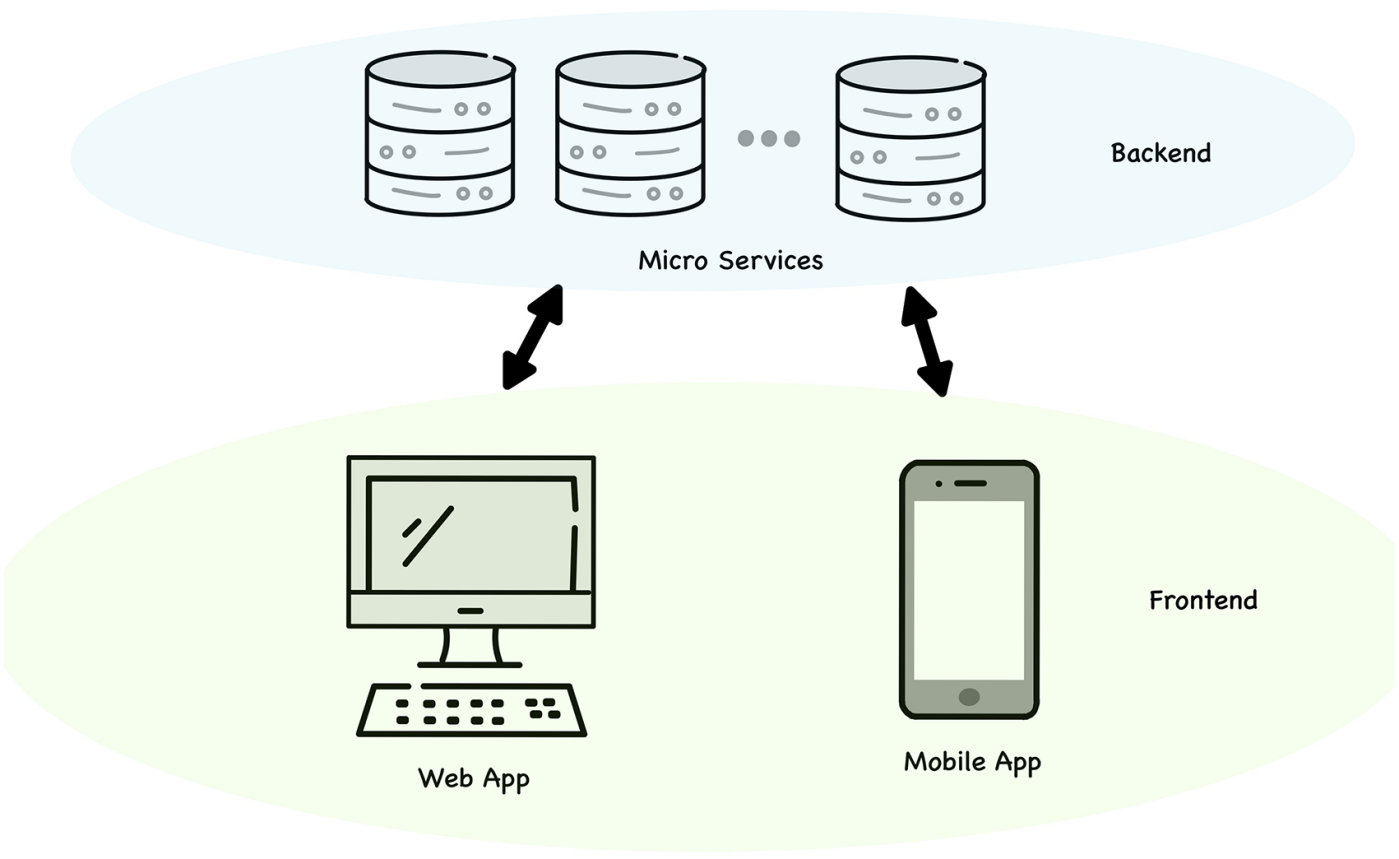
MOST platform.

In addition, COVID-19 pandemic has transformed the context for e-health technologies (Torous et al., 2021). During the Covid-19 outbreak, several countries employed various digital health strategies to control the pandemic course. These digital health strategies, especially mobile health technology, significantly support health care systems by promoting self-surveillance, self-monitoring, self-efficacy, and high quality freely available resources (Fagherazzi et al., 2020; Inkster et al., 2020; Kalhori et al., 2021). Finally, resorting to mobile health technology might constitute a wide dissemination tool of treatment information and mental health preventive strategies (Green et al., 2021; Figure 1).

## Rationale

When lockdowns began in early 2020, few of us imagined the long road ahead. The Covid-19 pandemic shed light on our vulnerabilities. In fact, we believe that the post-pandemic period is a fruitful moment to analyze and increase awareness regarding emotions, thoughts, and behaviors emerged during the pandemic (Maggi et al., 2022). Pitel and Ballová Mikušková (2021) highlighted the need to explore the development of cognitive distortions and their stability during prolonged health threatening situations (i.e., Covid-19 pandemic), in which negative and unwarranted beliefs predict maladaptive behaviors and psychological symptoms (Čavojová et al., 2020; Somma et al., 2020; Aureus et al., 2021; Pavela Banai et al., 2021; Teovanović et al., 2021).

With this in mind, we decided to adopt CBT techniques to help individuals transform maladaptive strategies into functional coping mechanisms to manage the post-pandemic “normality” and prevent the worsening of potential long-term Covid-19-induced psychological difficulties. Keeping in mind the potential benefits offered by e-health technologies, we adhere to the available literature and the WHO recommendations. In fact, mobile self-monitoring and self-management strategies could stimulate learning about personal mental health, empower individuals to assume a more active role in their health care, and promote the development of functional mental health strategies (Kenny et al., 2016; Beiwinkel et al., 2017; Inkster et al., 2020; Green et al., 2021). In fact, the use of mobile technologies in mental health has been encouraged by the World Health Organization (2021) to promote psychological treatment and self-monitoring of mental health, providing tailored feedback to support a positive change. In addition, self-management seems to help people to identify the need for clinical approaches, providing potentially preventive interventions (Karasouli and Adams, 2014; Taylor et al., 2020).

### Objectives

The direct and indirect psychological and social effects of the Covid-19 pandemic are pervasive, affecting individuals’ mental health now and in the future (Holmes et al., 2020; Mahmud et al., 2021). Therefore, the successful use of quarantines and other preventive measures on behalf of public health requires reducing, as far as possible, the negative effects associated with it, including psychological distress (Brooks et al., 2020). Furthermore, the presence of Covid-19-related cognitive distortions shows the need for early intervention, which is a primary concern in health care (Aureus et al., 2021).

The present paper describes the protocol of the Mobile Opportunity against Stress (MOST) study, which will aim to test the effectiveness, in the post-pandemic era, of a mobile platform for stress self-management adapted to the needs of specific vulnerable populations: young adults, older individuals, frontline and non-frontline healthcare professionals, and entrepreneurs. These groups were selected as representatives of populations characterized by a different level of contagion risk and, therefore, exposed to a different level of stress. The potential differences in the mobile app usage and effectiveness between the specific vulnerable populations recruited will also be explored. The mobile app will help at monitoring personal Covid-19-related negative emotions and distress, raising awareness and knowledge about the pandemic course and the health-promoting behavior’s, and enhancing functional coping strategies and self-efficacy beliefs to reduce Covid-19 related stress and psychological difficulties.

### Trial design

The effectiveness of the smartphone app will be investigated through a single-group longitudinal research design. No control group will be included due to the open trial design of the study.

## Materials and methods

### Participants

#### Study setting

Participants will be identified and enrolled in the general population of the Tuscany region, in Italy. The frontline and non-frontline healthcare professional groups will be identified by health workers belonging to any health professional category working in Covid-19 units and in other units in Tuscany (i.e., any other type of healthcare setting, working with any type of patients and any type of pathological condition). The university students will be identified and recruited among the undergraduate populations of the Tuscany Universities of Siena, Florence, and Pisa. Older people (aged ≥65 years old) will be identified and recruited in different contexts in Tuscany including associations for leisure activities of senior citizens, community services and volunteer associations for older people in Tuscany. Older people will be considered eligible if they are not hospitalized in any healthcare facility or a nursing home. The entrepreneurs/dealers will be identified and recruited among those who operate in Tuscany in the economic sectors most affected by the pandemic such as the tourism and hospitality sectors (i.e., hotels) and commercial establishments including fitness industries (e.g., gyms, beauty centers) which were forced to close during some of the phases of the pandemic and still are affected by the extremely slow recovery of the economy.

#### Eligibility criteria

Individuals will be included in the study if: (a) they are adults residing in Tuscany (aged ≥18 year old), (b) they belong to one of the risk above mentioned groups of the general population (i.e., healthcare professionals working in Covid-19 units or other units, university students, older people, entrepreneurs/dealers), (c) they have a smartphone with an Internet connection and are able to use it and/or have experience in the use of smartphone applications, (d) they can read written texts in Italian language, I they do not have any self-reported medical or neuropsychiatric conditions which can interfere with the use of a smartphone app or can make it difficult to understand the materials contained in the app (e.g., intellectual disabilities, sensory deficits, neurological pathologies, severe psychiatric conditions in the acute phase such as an ongoing acute depressive, bipolar or psychotic episode).

### Intervention

#### Intervention description

Participants will be allowed to use the contents of the app daily for a total period of four months from the baseline assessment, according to a frequency and duration at their discretion within this period. The app will contain written and audio-visual materials inspired by evidence-based psychoeducation and cognitive behavioral exercises and techniques, which have already been demonstrated to be effective in promoting well-being and preventing stress by randomized controlled trials and meta-analyses (Spek et al., 2007; Carlbring et al., 2011; Simon et al., 2019; Kladnitski et al., 2020).

App material will consist of (1) psychoeducation readings and audio-visual materials about emotions and the so-called cognitive distortions (i.e., mental traps that act as factors of vulnerability and maintenance of psychological stress), (2) (ABC, “*Activating Event – Beliefs – Consequences*”) diaries of recording of automatic thoughts, emotions and behavior’s (an example of an ABC diary completed by a fictional healthcare worker is presented in Table 1), (3) cognitive restructuring cards for the identification of cognitive distortions and modification of negative automatic thoughts, (4) problem-solving cards applied to daily critical situations, (5) audio exercises for meditation and distancing from automatic thoughts for the management of negative emotions, (6) factsheets on the construction of personal values. The examples of the diary and problem-solving cards applied to daily critical situations will be different for each target group and tailored to their needs and daily experiences. These materials, indeed, will be adapted to the stress-related aspects of the pandemic (e.g., perception of vulnerability, benefits and costs of preventive actions, negative consequences of social distancing in daily life, measures to deal with the pandemic on a daily basis, economic gain) emerged through focus groups conducted online with representatives of the included populations.

**Table 1:** Example of an ABC daily diary completed by a fictional healthcare worker (Mario).

**ACTIVATING EVENT** *Where? When? What?*	**MY AUTOMATIC THOUGHTS** *What was going through my mind?*	**MY FEELINGS** *How did I feel?*	**MY PHYSICAL SENSATIONS** *Where I was feeling those sensations in my body*	**MY BEHAVIOURS** *What I did in response to thoughts, feelings and physical sensations*
***MARIO***	*Too many visits today*	*Anger*	*Fatigue*	*Try to answer patients’ questions, meet their requests as much as possible and be concentrated over job tasks*
***(50 years old, family physician)***	*I am not prepared to cope with this stressful situation… alone on the frontline*	*Helplessness*	*Headache*	*Complain about colleagues*
*During the last months since the pandemic beginning, there has been an increased number of visits, patients seeking help, phone calls at office*	*Even tonight, when I get home, I could infect my family members*	*Anxiety*	*Muscle tension*	*Extend the work shifts*
		*Sense of guilt*	*Shortness of breath*	*Avoid rest, family life and leisure time*
				*Worry every night*

#### Strategies to improve adherence to interventions

One of the main issues regarding self-management interventions delivered by mobile apps is the risk of sub-optimal adherence, i.e., a minimal or less frequent use of the app. Such utilization of the application could limit the effectiveness of the intervention. To enhance participation and a regular use of our app, users will be guided in the compilation of the diary by an easy-to-follow step-by-step process and a reminder will be sent to them through mobile notification and email. Moreover, during the phase of data analysis, sub-optimal adherence will be controlled by including the frequency of use of the app (measured with the number of weekly accesses and mean duration of accesses) as a covariate in the ANCOVA model.

Another issue that mobile apps must face concerns the risk of relatively high drop-out rates. In a recent meta-analysis of randomized controlled trials conducted in adult samples, Linardon and Fuller-Tyszkiewicz (2020) found a drop-out rate of 24.1% at short-term follow up and 35.5% at longer-term follow up for mobile applications with rates varying according to target mental health conditions. In another meta-analysis (Torous et al., 2020), drop-out rates could reach 48%, when accounting for publication bias, in apps targeting participants with depressive symptoms. Basing on this evidence, we expect a similar range of dropout rates for our sample.

### Outcomes

The effectiveness of the mobile app will be evaluated on the following outcomes: (i) perceived susceptibility and severity of the pandemic situation, perceived benefits, and costs of preventive health behavior’s (such as using a mask), (ii) knowledge about Covid-19 preventive behavior’s and negative consequences of social distancing, (iii) stress and psychopathological symptoms (i.e., anxiety, depression, and post-traumatic stress symptoms) and cognitive distortions.

These variables will be measured at a baseline assessment (i.e., each participant will complete the questionnaires within 2 weeks before using the app), at an intermediate evaluation (i.e., 2 months after the baseline evaluation), at a post-test evaluation (i.e., 4 months after the baseline evaluation), and at a follow-up evaluation conducted 6 months after the baseline assessment. The endpoint consists of the variation in depressive, anxious and stress states, cognitive distortions, and coping strategies, from the baseline to the intermediate, post-test and follow-up evaluations. The scores in all questionnaires that measure the endpoints will be aggregated in the form of mean scores with relative standard deviations at every evaluation. No harm derived by the use of the self-help application is expected.

#### Participant timeline

Table 2 shows the participant timeline.

**Table 2:** Participant timeline.

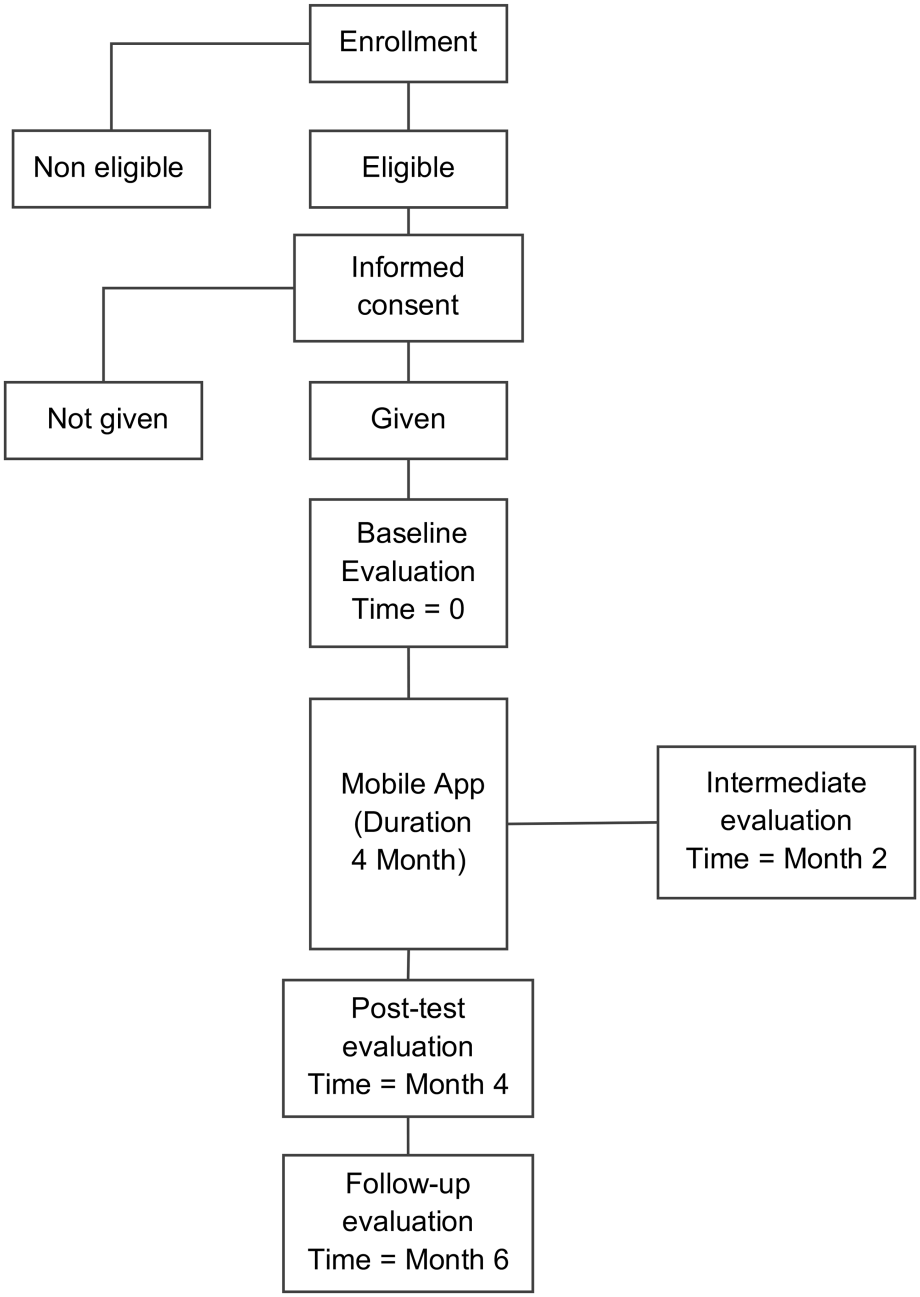

#### Sample size

According to an *a-priori* power analysis, at least 1,800 participants are required to conduct a repeated measures ANCOVA with between-within factors and interactions to estimate a medium effect size on each one of the outcomes with 95% power and a significance level set at 0.05.

#### Recruitment

Participants’ enrolment will last for 2 months. During participants’ recruitment, a series of meetings will be organized, respectively in the healthcare services, universities, tourism and fitness industry associations, older people’s associations, to disseminate the study’s aims, and encourage participation. A website will be created to disseminate the study’s aims and online brochures about the study will be sent to the email addresses of Department directors of healthcare services, representatives of university students, representatives of industry associations and representatives of elderly associations, respectively. Eligible participants will be emailed the link through which they can download the smartphone application.

### Data collection, management, and analysis

#### Plans for assessment and collection of outcomes

All the data will be collected through the mobile application: each participant will be invited to compile the questionnaire at the above mentioned timepoints. Outcomes will be measured by the scores on the following psychometric scales: Depression Anxiety Stress Scale-21 (DASS-21; Lovibond and Lovibond, 1996), Cognitive Distortions Scale (CDS; Covin et al., 2011) and Coping Orientations to Problems Experiences -New Italian Version (COPE-NVI-25; Foà et al., 2015). An overview of the study tools is presented in Table 3.

**Table 3:** Study tools.

**Measure**	**Construct and item**	**Psychometric proprieties**
Depression anxiety stress scale-21 (DASS-21; Lovibond and Lovibond, 1996)	It measures stress, anxiety and depression symptoms. 21 items.	Good convergent and discriminant validity and high internal consistency in English (Daza et al., 2002; Norton, 2007) and Italian (Bottesi et al., 2015) samples.
Cognitive distortions scale (CDS; Covin et al., 2011)	It measures the frequency of cognitive distortions in two domains: interpersonal and personal achievement. 20 items.	Excellent overall reliability in English (Covin et al., 2011) and Italian (Aldi et al., 2020) samples.
Coping orientation to problems experiences – New Italian version (COPE-NVI-25; Foà et al., 2015)	It assesses the coping styles in difficult or stressful situations. 25 items.	Good to excellent reliability across the scales in the Italian sample Foà et al. (2015).
Self-report questionnaire related to the health belief model	The perception of vulnerability/severity with respect to the Covid-19. The perception of the benefits/disadvantages of health/preventive behaviors with respect to the	

The DASS-21 is a 21-item questionnaire that measures one-week state negative affect, developed with the specific aim of achieving maximal differentiation between the affective symptoms of depression, anxiety, and tension/stress. Respondents indicate the extent to which they experienced each of the symptoms depicted in the items during the previous week on a 4-point Likert-type scale between 0 (*Did not apply to me at all*) and 3 (*Applied to me very much, or most of the time*). The factor structure of the DASS-21 is stable, and its scales showed good convergent and discriminant validity and high internal consistency in clinical and in non-clinical samples and in different ethnic groups in adults (Daza et al., 2002; Norton, 2007). The Italian version (Bottesi et al., 2015) demonstrated good internal consistency.

The CDS (Covin et al., 2011) is a measure that individually assesses the frequency with which respondents experience 10 cognitive errors in interpersonal and achievement domains. Covin et al. (2011) examined the validity and reliability of the scale in a non-clinical sample and found an excellent overall reliability (*α* = 0.85), acceptable reliability coefficients for the two subscales (Interpersonal subscale, *α* = 0.75; Achievement subscale, *α* = 0.79). The Italian version (Aldi et al., 2020) showed a single factor with excellent reliability (*α* = 0.96).

The COPE-NVI-25 was developed by Foà et al. (2015) with the aim to assess the coping styles adopted by subjects in difficult or stressful situations. This is a short version of the COPE (Carver et al., 1989). The COPE-NVI-25 showed as good psychometric skills as those shown by the original version. It is capable to evaluate coping strategies in a hospital context, where a simple and fast assessment is often required; it is proved to be an instrument as valid as the original COPE, but easier to administer. The COPE-NVI-25 is a multi-dimensional inventory that assesses individual differences in coping styles. It is comprised of 25 items, which are rated on a 4-point scale ranging from 1 (I usually do not do this at all) to 4 (I usually do this a lot). The instrument includes five subscales corresponding to five different coping styles: Social Support (example item: “*I seek moral support from friends and relatives*”), Avoidance Strategies (example item: “*I admit to myself that I cannot deal with it, and quit trying*”), Positive Attitude (example item: “*I try to learn something good from experience*”), Problem Solving (example item: “*I focus on dealing with this problem and, if necessary, I put from other things aside*”), and Turning to Religion (example item: “*I try to find comfort in my religion*”). A higher score on a particular subscale indicates a greater use of that specific coping strategy. In the present study, the scores for each one of the COPE-NVI-25 scales were calculated by summing the scores obtained by the participants on the items that were related to the target scale in the validation study. The reliability indices were good to excellent across all the COPE-NVI-25 scales (Social Support scale: Cronbach’s alpha = 0.80; Avoidance Strategies scale: Cronbach’s alpha = 0.72; Positive Attitude scale: Cronbach’s alpha = 0.90; Problem Solving scale: Cronbach’s alpha = 0.86; Turning to Religion scale: Cronbach’s alpha = 0.96).

Additional endpoints include the scores on a self-report questionnaire that will address the following areas: the perception of vulnerability/severity with respect to the Covid-19 infection (example item: “*How much risk do you feel about getting Covid-19 infection?*), the perception of the benefits/disadvantages of health/preventive behavior’s with respect to the Covid-19 infection (example items: *“How beneficial were health/preventive behaviours?”; “How disadvantageous?”*).

#### Plans to promote participant retention and complete follow-up

The patients will receive extensive information about the study set-up and requirements during the recruitment. The importance of completion of the follow-up will be stressed. Patients are allowed to stop at any time during the study and are not obliged to give a reason to discontinue. If possible, the patient will be asked to complete the survey at 2, 4 and 6 months after inclusion. Questionnaires are completed using the mobile app, and therefore participants can do this at any convenient moment. All patients are reminded throughout mobile app notifications to fill out the questionnaires. Throughout the follow-up period, the researchers will check responses and if necessary, contact participants for completion of their follow-up.

#### Data management

Data collected through the app will be entered in a private dataset hosted on a secure university-based server. Two independent researchers will perform the analysis to control for potential errors.

#### Statistical methods for primary and secondary outcomes

Normality of the variables will be detected by Kolmogorov–Smirnov test. A series of repeated measures ANCOVAs will be performed to assess the effectiveness of the smartphone app on the outcomes. The baseline scores on the scales will be entered in the statistical model as covariates, the type of target group as a factor and the scores for each timepoint as dependent variables, respectively. Effect sizes will be calculated as Squared Eta indices (η^2^) according to the formula provided by Olejnik and Algina (2003). Following Cohen et al. (1998), effect sizes of 0.01, 0.06, and 0.14 will be interpreted as small, medium, and large, respectively. The statistical significance will be set at *p* < 0.05.

#### Methods in analysis to handle protocol non-adherence and any statistical methods to handle missing data

Missing baseline data will be treated by multiple imputation (Sinharay et al., 2001). Missing longitudinal data will be managed through an intention-to-treat analysis with the last observation carried forward technique (Newell, 1992). The statistical analyses will be carried out by the software SPSS version 25.00.

### Monitoring

#### Composition of the coordinating center and trial steering committee

This is a multicenter study designed and coordinated in the Department of Medical Sciences, Surgery and Neurosciences, University of Siena. Day to day support for the trial is provided by:

Principle investigator: takes supervision of the trial. Data managers: organizes data capture, safeguards quality and data. Study coordinators: coordinates study phases, annual safety reports.

The study team meets every month to monitor the study progress and the potential pitfalls. There is no trial steering committee or stakeholder and public involvement group. The decision to terminate the study is up to the principal investigator.

#### Adverse event reporting and harms

All adverse events reported by the subject will be recorded and handled by the principal investigator.

#### Auditing

The Ethical Committee of the “Azienda Ospedaliera Universitaria Senese” will independently carry out interim and final monitoring of the study. The Funding Body, that is the Tuscany Region, has carried out (i) a first mid-term audit of the progress of the works with respect to the schedule and the milestones achieved, (ii) a financial statement on the reporting of expenses, and (iii) a special interim report on the activities carried out 1 year after the start of the project. A final verification by the Region on the same parameters will be made at the end of the project.

## Ethics and dissemination

The research protocol of this study was approved by the Ethical Committee of the “Azienda Ospedaliera Universitaria Senese” (Italy) on 28th October 2021 (Approval code: 20831). The Ethical Committee will be formally informed of any changes to the protocol. According to Wies et al. (2021), digital health technologies provide technical, scientific, ethical, and regulatory challenges. Despite the centrality of data collection for study purposes and for the administration of the platform, the users’ privacy and security need to be assured, so all the private information will be anonymous. Participants will be informed of the data management policy and anonymity will be guaranteed before obtaining informed consent.

### Dissemination policy

The study results will be published in a peer-reviewed journal regardless of the result. Additionally, participation and presentations at relevant national and international academic conventions will promote dissemination. Patients will receive a summary of the results in case they opted-in to receive outcomes on a study level.

## Anticipated results

The results of this study will provide helpful knowledge and insights into the effects of using an app for self-monitoring and managing long-term stress symptoms during pandemic periods. Self-management methods can be a resource to enable people to deal with stressful events, to discover external/internal resources, to mobilize them and to promote effective coping strategies in a health-promoting manner. Indeed, supporting people’s involvement in their care to identify problems early, also promotes more timely initiation of necessary treatment. Data obtained from the small pilot sample of users through direct observation, data collection through the application, and questionnaire administration will be triangulated to enhance the next application version. More specifically, the repeated measures from the baseline assessment to the follow-up evaluation will allow to identify potential critical points that might need to be improved or modified in the following app implementation. Furthermore, users’ responses to written and audio-visual psychoeducation materials usage, ABC diaries, cognitive restructuring and problem-solving cards, meditation and distancing from automatic thoughts exercises, and factsheets on the construction of personal values will provide useful information for the final app improvement. Moreover, these contents might help users to self-manage and self-monitor not just COVID-19-related stress, but stress in general and psychological long-term consequences, such as depression and anxiety. For example, the ABC diary used to face specific pandemic-related situations could be employed also in everyday life stressful conditions.

Our study samples will be large and heterogenous and will include subjects with different ages: from young adults over 18 years to elderly individuals. So, the mobile platform for stress-self management will not be restricted for use and application with the target vulnerable populations, but it can also be transferred to other populations and contexts. If successful, we anticipate the platform could have clinical benefits to various groups by providing symptom monitoring and early intervention and decreasing barriers to treatment-seeking. In fact, the population-level approach has the potential to improve mental health outcomes in pandemic periods for many people. If this protocol is proven effective, it can be expanded to any population at risk of experiencing mental distress during a continuous public health emergency such as the Covid-19 pandemic.

Moreover, if effective, this application could also be implemented in other similar contexts in which stress management could be particularly helpful like in the business and school sectors. Non-healthcare workers, high school students, and teachers present similar characteristics to those of our population and could benefit from an app targeting.

Although the current protocol is addressed solely to Italian-speaking individuals, a future potential expansion could involve the integration of facilitators to reach underserved people from other ethnic groups.

### Trial status

The current protocol is version 1 of 04-11-2022. It is estimated that patient recruitment will begin in February 2023 and will be completed around September 2023. Currently, we are approaching the pilot phase of the study that will begin in December 2022.

## Ethics statement

The studies involving human participants were reviewed and approved by Ethical Committee of the University Hospital of Siena. Approval code: 20831. The patients/participants provided their written informed consent to participate in this study.

## Author contributions

FI, DP and NA have been involved in drafting and revising the manuscript critically and have given final approval of the version to be published. AP: conceptualization, funding acquisition, investigation, project administration, and writing-original draft preparation. BG: investigation, resources, and writing—original draft preparation. DB: software and writing—original draft preparation. AF: software and writing—review and editing. GM: conceptualization, project administration, resources, and writing-original draft preparation. GG and LL: resources and writing—review and editing. FG: investigation, formal analysis, and writing—original draft preparation. SB: investigation, formal analysis, and writing—original draft preparation. GF: resources and writing-original draft preparation. AC and FF: supervision and writing – review and editing. SC: conceptualization, project administration, supervision, and writing – review and editing.

## Funding

This research is funded by Bando Salute Regione Toscana. This is an investigator initiated clinical trial.

## Conflict of interest

The authors declare that the research was conducted in the absence of any commercial or financial relationships that could be construed as a potential conflict of interest.

## Publisher’s note

All claims expressed in this article are solely those of the authors and do not necessarily represent those of their affiliated organizations, or those of the publisher, the editors and the reviewers. Any product that may be evaluated in this article, or claim that may be made by its manufacturer, is not guaranteed or endorsed by the publisher.
